# Tetra-μ-acetato-κ^8^ *O*:*O*′-bis­[(2-phen­oxy­pyrimidine-κ*N* ^1^)copper(II)](*Cu*—*Cu*)

**DOI:** 10.1107/S1600536811044345

**Published:** 2011-10-29

**Authors:** Zainal Abidin Fairuz, Zanariah Abdullah, Shah Bakhtiar Nasir, Seik Weng Ng, Edward R. T. Tiekink

**Affiliations:** a Department of Chemistry, University of Malaya, 50603 Kuala Lumpur, Malaysia; b Chemistry Department, Faculty of Science, King Abdulaziz University, PO Box 80203 Jeddah, Saudi Arabia

## Abstract

The complete dinuclear mol­ecule of the title complex, [Cu_2_(CH_3_COO)_4_(C_10_H_8_N_2_O)_2_], is generated by a centre of inversion. The Cu^II^ atom is in a distorted octa­hedral coordination geometry defined by four O atoms derived from four bridging acetate ligands, a terminally connected pyrimidine N atom and a Cu atom.

## Related literature

For structures of related examples of tetra­kis­acetato­bis­[(*N*-donor)copper] complexes, see: Fairuz *et al.* (2010*a* ▶,*b* ▶).
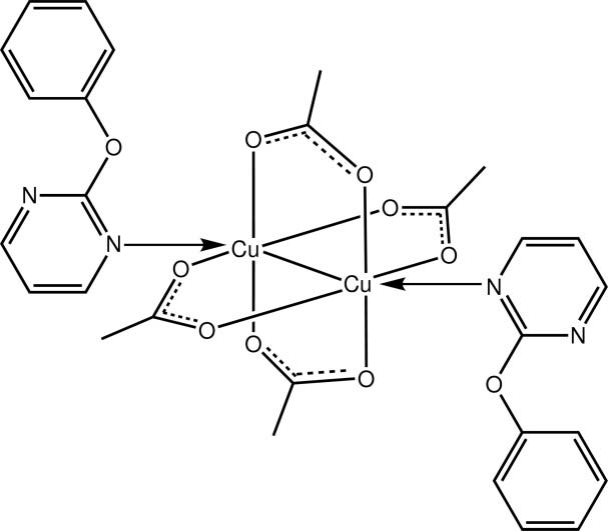


## Experimental

### 

#### Crystal data

[Cu_2_(C_2_H_3_O_2_)_4_(C_10_H_8_N_2_O)_2_]
*M* *_r_* = 707.64
Monoclinic, 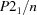

*a* = 11.0738 (9) Å
*b* = 7.5002 (6) Å
*c* = 18.0539 (14) Å
β = 100.579 (1)°
*V* = 1474.0 (2) Å^3^
*Z* = 2
Mo *K*α radiation
μ = 1.51 mm^−1^
*T* = 100 K
0.25 × 0.24 × 0.04 mm

#### Data collection

Bruker SMART APEX diffractometer
Absorption correction: multi-scan (*SADABS*; Sheldrick, 1996 ▶) *T*_min_ = 0.637, *T*_max_ = 0.746
11124 measured reflections
2581 independent reflections
2249 reflections with *I* > 2σ(*I*)
*R*_int_ = 0.037

#### Refinement

*R*[*F* ^2^ > 2σ(*F* ^2^)] = 0.056
*wR*(*F* ^2^) = 0.175
*S* = 1.08
2581 reflections
201 parameters
H-atom parameters constrained
Δρ_max_ = 1.83 e Å^−3^
Δρ_min_ = −0.55 e Å^−3^

### 

Data collection: *APEX2* (Bruker, 2009 ▶); cell refinement: *SAINT* (Bruker, 2009 ▶); data reduction: *SAINT*; program(s) used to solve structure: *SHELXS97* (Sheldrick, 2008 ▶); program(s) used to refine structure: *SHELXL97* (Sheldrick, 2008 ▶); molecular graphics: *ORTEP-3* (Farrugia, 1997 ▶) and *DIAMOND* (Brandenburg, 2006 ▶); software used to prepare material for publication: *publCIF* (Westrip, 2010 ▶).

## Supplementary Material

Crystal structure: contains datablock(s) global, I. DOI: 10.1107/S1600536811044345/hg5123sup1.cif

Structure factors: contains datablock(s) I. DOI: 10.1107/S1600536811044345/hg5123Isup2.hkl

Additional supplementary materials:  crystallographic information; 3D view; checkCIF report

## Figures and Tables

**Table 1:** Selected bond lengths (Å)

Cu1—O3	1.954 (4)
Cu1—O1	1.966 (4)
Cu1—O4^i^	1.977 (3)
Cu1—O2^i^	1.979 (4)
Cu1—N1	2.207 (4)
Cu1—Cu1^i^	2.6154 (10)

Symmetry code: (i) 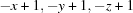.

**Fig. 1.**
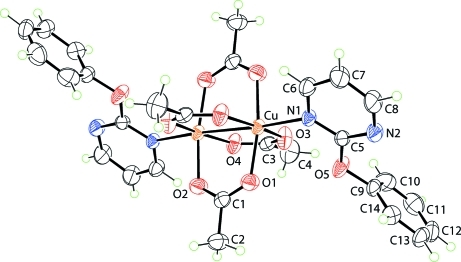
The molecular structure of (I) showing the atom-labelling scheme and displacement ellipsoids at the 50% probability level. The complex is centrosymmetric. The unlabelled atoms are related by the symmetry operation 1 - x, 1 - y, 1 - z.

## supplementary crystallographic information

### Comment

It was in connection with recent structural studies of tetrakisacetatobis[(*N*-donor)copper(II)] complexes (Fairuz *et al.*, 2010*a*; Fairuz *et al.*, 2010*b*), that the crystal structure of the title complex, (I), was investigated. The complex, Fig. 1, is centrosymmetric and features four symmetrically bridging acetate ligands and two terminally connected N atoms from the 2-phenoxypyrimidine ligands, Table 1. The Cu—Cu distance is 2.6154 (10) Å. The resulting CuNO_4_ donor set defines a distorted octahedral geometry. The 2-phenoxypyrimidine ligand is twisted with the dihedral angle between the pyrimidyl and benzene rings being 63.3 (3)°.

### Experimental

2-Phenoxypyrimidine (1.1 mmol) was dissolved in acetonitrile (15 ml), added to trimethyl orthoformate (10 ml) and the mixture then heated to 50°C. Copper acetate (0.5 mmol) dissolved in acetonitrile (15 ml) was added to the solution. The green precipitate that formed was collected and recrystallized from acetonitrile to give green crystals.

### Refinement

Hydrogen atoms were placed at calculated positions (C—H 0.95–0.98 Å) and were treated as riding on their parent carbon atoms, with *U*(H) set to 1.2–1.5 times *U*_eq_(C). The maximum and minimum residual electron density peaks of 1.83 and 0.55 e Å^-3^, respectively, were located 1.42 Å and 0.44 Å from the H6 and Cu atoms, respectively.

### Crystal data

**Table:**

[Cu_2_(C_2_H_3_O_2_)_4_(C_10_H_8_N_2_O)_2_]	*F*(000) = 724
*M**_r_* = 707.64	*D*_x_ = 1.594 Mg m^−^^3^
Monoclinic, *P*2_1_/*n*	Mo *K*α radiation, λ = 0.71073 Å
Hall symbol: -P 2yn	Cell parameters from 3884 reflections
*a* = 11.0738 (9) Å	θ = 2.3–27.1°
*b* = 7.5002 (6) Å	µ = 1.51 mm^−^^1^
*c* = 18.0539 (14) Å	*T* = 100 K
β = 100.579 (1)°	Plate, green
*V* = 1474.0 (2) Å^3^	0.25 × 0.24 × 0.04 mm
*Z* = 2	

### Data collection

**Table:**

Bruker SMART APEX diffractometer	2581 independent reflections
Radiation source: fine-focus sealed tube	2249 reflections with *I* > 2σ(*I*)
graphite	*R*_int_ = 0.037
ω scans	θ_max_ = 25.0°, θ_min_ = 2.0°
Absorption correction: multi-scan (*SADABS*; Sheldrick, 1996)	*h* = −13→13
*T*_min_ = 0.637, *T*_max_ = 0.746	*k* = −8→8
11124 measured reflections	*l* = −21→21

### Refinement

**Table:**

Refinement on *F*^2^	Primary atom site location: structure-invariant direct methods
Least-squares matrix: full	Secondary atom site location: difference Fourier map
*R*[*F*^2^ > 2σ(*F*^2^)] = 0.056	Hydrogen site location: inferred from neighbouring sites
*wR*(*F*^2^) = 0.175	H-atom parameters constrained
*S* = 1.08	*w* = 1/[σ^2^(*F*_o_^2^) + (0.1094*P*)^2^ + 3.359*P*] where *P* = (*F*_o_^2^ + 2*F*_c_^2^)/3
2581 reflections	(Δ/σ)_max_ = 0.001
201 parameters	Δρ_max_ = 1.83 e Å^−^^3^
0 restraints	Δρ_min_ = −0.55 e Å^−^^3^

### Special details

**Table:**

Geometry. All e.s.d.'s (except the e.s.d. in the dihedral angle between two l.s. planes) are estimated using the full covariance matrix. The cell e.s.d.'s are taken into account individually in the estimation of e.s.d.'s in distances, angles and torsion angles; correlations between e.s.d.'s in cell parameters are only used when they are defined by crystal symmetry. An approximate (isotropic) treatment of cell e.s.d.'s is used for estimating e.s.d.'s involving l.s. planes.

### Fractional atomic coordinates and isotropic or equivalent isotropic displacement parameters (Å^2^)

**Table:**

	*x*	*y*	*z*	*U*_iso_*/*U*_eq_	
Cu1	0.41133 (5)	0.58752 (7)	0.52267 (3)	0.0279 (3)	
O1	0.3059 (3)	0.4099 (4)	0.4621 (2)	0.0415 (9)	
O2	0.4556 (3)	0.2595 (5)	0.4231 (2)	0.0463 (9)	
O3	0.4384 (4)	0.4244 (5)	0.6085 (2)	0.0479 (10)	
O4	0.5873 (3)	0.2712 (5)	0.5697 (2)	0.0421 (9)	
O5	0.1716 (4)	0.5271 (5)	0.5969 (3)	0.0520 (11)	
N1	0.2668 (3)	0.7572 (5)	0.5552 (2)	0.0304 (8)	
N2	0.0888 (4)	0.8062 (6)	0.6078 (3)	0.0436 (11)	
C1	0.5176 (4)	0.3045 (6)	0.6154 (3)	0.0326 (10)	
C2	0.5298 (7)	0.1861 (9)	0.6841 (3)	0.0597 (17)	
H2A	0.6101	0.2059	0.7163	0.090*	
H2B	0.5228	0.0609	0.6683	0.090*	
H2C	0.4646	0.2146	0.7122	0.090*	
C3	0.3455 (5)	0.2911 (6)	0.4241 (3)	0.0331 (10)	
C4	0.2508 (5)	0.1755 (8)	0.3750 (3)	0.0484 (14)	
H4A	0.2681	0.0497	0.3872	0.073*	
H4B	0.2545	0.1964	0.3218	0.073*	
H4C	0.1687	0.2056	0.3841	0.073*	
C5	0.1732 (4)	0.7050 (6)	0.5869 (3)	0.0316 (10)	
C6	0.2763 (5)	0.9324 (7)	0.5446 (3)	0.0413 (13)	
H6	0.3425	0.9762	0.5231	0.050*	
C7	0.1930 (6)	1.0506 (8)	0.5640 (4)	0.0546 (16)	
H7	0.1998	1.1753	0.5565	0.065*	
C8	0.0997 (6)	0.9796 (8)	0.5947 (4)	0.0565 (16)	
H8	0.0394	1.0582	0.6075	0.068*	
C9	0.0924 (5)	0.4472 (6)	0.6393 (3)	0.0374 (12)	
C10	0.1489 (5)	0.3652 (9)	0.7058 (3)	0.0515 (14)	
H10	0.2348	0.3764	0.7234	0.062*	
C11	0.0762 (6)	0.2666 (9)	0.7457 (3)	0.0567 (16)	
H11	0.1127	0.2093	0.7913	0.068*	
C12	−0.0480 (6)	0.2509 (8)	0.7200 (3)	0.0487 (14)	
H12	−0.0970	0.1818	0.7473	0.058*	
C13	−0.1003 (5)	0.3352 (8)	0.6553 (3)	0.0461 (13)	
H13	−0.1864	0.3255	0.6383	0.055*	
C14	−0.0313 (5)	0.4344 (7)	0.6136 (3)	0.0405 (12)	
H14	−0.0690	0.4921	0.5684	0.049*	

### Atomic displacement parameters (Å^2^)

**Table:**

	*U*^11^	*U*^22^	*U*^33^	*U*^12^	*U*^13^	*U*^23^
Cu1	0.0235 (4)	0.0277 (4)	0.0343 (4)	0.0048 (2)	0.0105 (2)	0.0017 (2)
O1	0.0300 (19)	0.040 (2)	0.054 (2)	0.0005 (14)	0.0077 (17)	−0.0082 (16)
O2	0.0293 (18)	0.048 (2)	0.062 (2)	−0.0022 (16)	0.0099 (16)	−0.0188 (19)
O3	0.052 (2)	0.052 (2)	0.044 (2)	0.0165 (18)	0.0223 (19)	0.0130 (17)
O4	0.043 (2)	0.0395 (19)	0.049 (2)	0.0127 (16)	0.0212 (17)	0.0127 (16)
O5	0.046 (2)	0.0279 (18)	0.094 (3)	0.0067 (16)	0.045 (2)	0.0048 (19)
N1	0.0276 (19)	0.0273 (19)	0.039 (2)	0.0041 (15)	0.0134 (16)	0.0005 (17)
N2	0.039 (2)	0.033 (2)	0.064 (3)	0.0047 (18)	0.025 (2)	−0.006 (2)
C1	0.032 (2)	0.031 (2)	0.035 (2)	−0.005 (2)	0.005 (2)	0.0059 (19)
C2	0.067 (4)	0.064 (4)	0.049 (3)	0.010 (3)	0.013 (3)	0.026 (3)
C3	0.035 (3)	0.033 (2)	0.031 (2)	−0.002 (2)	0.005 (2)	0.0073 (19)
C4	0.040 (3)	0.048 (3)	0.055 (3)	−0.008 (2)	0.002 (3)	−0.006 (3)
C5	0.025 (2)	0.029 (2)	0.042 (3)	0.0016 (18)	0.0096 (19)	−0.0058 (19)
C6	0.038 (3)	0.035 (3)	0.055 (3)	0.002 (2)	0.020 (3)	0.001 (2)
C7	0.068 (4)	0.031 (3)	0.072 (4)	0.007 (3)	0.033 (3)	0.001 (3)
C8	0.059 (4)	0.032 (3)	0.089 (5)	0.013 (3)	0.041 (3)	−0.007 (3)
C9	0.035 (3)	0.028 (2)	0.054 (3)	0.002 (2)	0.023 (2)	−0.004 (2)
C10	0.036 (3)	0.058 (3)	0.055 (4)	0.002 (3)	−0.006 (3)	−0.007 (3)
C11	0.074 (4)	0.058 (4)	0.037 (3)	0.006 (3)	0.007 (3)	0.004 (3)
C12	0.055 (3)	0.044 (3)	0.055 (3)	0.001 (3)	0.032 (3)	0.001 (3)
C13	0.028 (3)	0.047 (3)	0.067 (4)	0.004 (2)	0.019 (2)	−0.003 (3)
C14	0.035 (3)	0.040 (3)	0.045 (3)	0.007 (2)	0.004 (2)	0.003 (2)

### Geometric parameters (Å, °)

**Table:**

Cu1—O3	1.954 (4)	C2—H2C	0.9800
Cu1—O1	1.966 (4)	C3—C4	1.515 (7)
Cu1—O4^i^	1.977 (3)	C4—H4A	0.9800
Cu1—O2^i^	1.979 (4)	C4—H4B	0.9800
Cu1—N1	2.207 (4)	C4—H4C	0.9800
Cu1—Cu1^i^	2.6154 (10)	C6—C7	1.371 (8)
O1—C3	1.252 (6)	C6—H6	0.9500
O2—C3	1.246 (6)	C7—C8	1.367 (9)
O2—Cu1^i^	1.979 (4)	C7—H7	0.9500
O3—C1	1.246 (6)	C8—H8	0.9500
O4—C1	1.254 (6)	C9—C14	1.367 (8)
O4—Cu1^i^	1.977 (3)	C9—C10	1.391 (8)
O5—C5	1.347 (6)	C10—C11	1.389 (9)
O5—C9	1.400 (6)	C10—H10	0.9500
N1—C5	1.331 (6)	C11—C12	1.373 (9)
N1—C6	1.335 (6)	C11—H11	0.9500
N2—C5	1.312 (6)	C12—C13	1.361 (8)
N2—C8	1.331 (8)	C12—H12	0.9500
C1—C2	1.511 (7)	C13—C14	1.383 (8)
C2—H2A	0.9800	C13—H13	0.9500
C2—H2B	0.9800	C14—H14	0.9500
			
O3—Cu1—O1	90.30 (18)	C3—C4—H4A	109.5
O3—Cu1—O4^i^	168.58 (15)	C3—C4—H4B	109.5
O1—Cu1—O4^i^	89.46 (16)	H4A—C4—H4B	109.5
O3—Cu1—O2^i^	88.77 (18)	C3—C4—H4C	109.5
O1—Cu1—O2^i^	168.63 (15)	H4A—C4—H4C	109.5
O4^i^—Cu1—O2^i^	89.22 (17)	H4B—C4—H4C	109.5
O3—Cu1—N1	99.39 (15)	N2—C5—N1	127.3 (4)
O1—Cu1—N1	98.78 (15)	N2—C5—O5	120.4 (4)
O4^i^—Cu1—N1	91.93 (14)	N1—C5—O5	112.3 (4)
O2^i^—Cu1—N1	92.55 (14)	N1—C6—C7	121.7 (5)
O3—Cu1—Cu1^i^	85.37 (11)	N1—C6—H6	119.1
O1—Cu1—Cu1^i^	83.66 (11)	C7—C6—H6	119.1
O4^i^—Cu1—Cu1^i^	83.26 (10)	C8—C7—C6	116.5 (5)
O2^i^—Cu1—Cu1^i^	84.97 (11)	C8—C7—H7	121.8
N1—Cu1—Cu1^i^	174.60 (11)	C6—C7—H7	121.8
C3—O1—Cu1	123.7 (3)	N2—C8—C7	123.5 (5)
C3—O2—Cu1^i^	121.6 (3)	N2—C8—H8	118.2
C1—O3—Cu1	122.2 (3)	C7—C8—H8	118.2
C1—O4—Cu1^i^	123.3 (3)	C14—C9—C10	121.7 (5)
C5—O5—C9	121.5 (4)	C14—C9—O5	122.3 (5)
C5—N1—C6	116.0 (4)	C10—C9—O5	115.7 (5)
C5—N1—Cu1	127.2 (3)	C11—C10—C9	118.1 (5)
C6—N1—Cu1	116.7 (3)	C11—C10—H10	120.9
C5—N2—C8	114.9 (5)	C9—C10—H10	120.9
O3—C1—O4	125.8 (4)	C12—C11—C10	120.7 (5)
O3—C1—C2	117.5 (5)	C12—C11—H11	119.6
O4—C1—C2	116.7 (5)	C10—C11—H11	119.6
C1—C2—H2A	109.5	C13—C12—C11	119.5 (5)
C1—C2—H2B	109.5	C13—C12—H12	120.3
H2A—C2—H2B	109.5	C11—C12—H12	120.3
C1—C2—H2C	109.5	C12—C13—C14	121.8 (5)
H2A—C2—H2C	109.5	C12—C13—H13	119.1
H2B—C2—H2C	109.5	C14—C13—H13	119.1
O2—C3—O1	125.9 (5)	C9—C14—C13	118.2 (5)
O2—C3—C4	117.1 (5)	C9—C14—H14	120.9
O1—C3—C4	117.0 (5)	C13—C14—H14	120.9
			
O3—Cu1—O1—C3	−87.8 (4)	Cu1—O1—C3—C4	−174.5 (4)
O4^i^—Cu1—O1—C3	80.8 (4)	C8—N2—C5—N1	0.3 (8)
O2^i^—Cu1—O1—C3	−2.6 (11)	C8—N2—C5—O5	−179.7 (6)
N1—Cu1—O1—C3	172.6 (4)	C6—N1—C5—N2	1.1 (8)
Cu1^i^—Cu1—O1—C3	−2.5 (4)	Cu1—N1—C5—N2	178.4 (4)
O1—Cu1—O3—C1	83.2 (4)	C6—N1—C5—O5	−178.8 (5)
O4^i^—Cu1—O3—C1	−5.6 (11)	Cu1—N1—C5—O5	−1.5 (6)
O2^i^—Cu1—O3—C1	−85.5 (4)	C9—O5—C5—N2	−9.6 (8)
N1—Cu1—O3—C1	−177.9 (4)	C9—O5—C5—N1	170.4 (5)
Cu1^i^—Cu1—O3—C1	−0.5 (4)	C5—N1—C6—C7	−1.2 (8)
O3—Cu1—N1—C5	−39.5 (4)	Cu1—N1—C6—C7	−178.8 (5)
O1—Cu1—N1—C5	52.2 (4)	N1—C6—C7—C8	0.0 (10)
O4^i^—Cu1—N1—C5	142.0 (4)	C5—N2—C8—C7	−1.8 (10)
O2^i^—Cu1—N1—C5	−128.7 (4)	C6—C7—C8—N2	1.7 (11)
O3—Cu1—N1—C6	137.7 (4)	C5—O5—C9—C14	72.4 (7)
O1—Cu1—N1—C6	−130.5 (4)	C5—O5—C9—C10	−114.0 (6)
O4^i^—Cu1—N1—C6	−40.8 (4)	C14—C9—C10—C11	0.9 (9)
O2^i^—Cu1—N1—C6	48.5 (4)	O5—C9—C10—C11	−172.8 (5)
Cu1—O3—C1—O4	−1.0 (8)	C9—C10—C11—C12	−0.1 (9)
Cu1—O3—C1—C2	−179.8 (4)	C10—C11—C12—C13	−0.9 (9)
Cu1^i^—O4—C1—O3	2.4 (7)	C11—C12—C13—C14	1.1 (9)
Cu1^i^—O4—C1—C2	−178.7 (4)	C10—C9—C14—C13	−0.7 (8)
Cu1^i^—O2—C3—O1	−5.4 (7)	O5—C9—C14—C13	172.5 (5)
Cu1^i^—O2—C3—C4	174.6 (4)	C12—C13—C14—C9	−0.3 (8)
Cu1—O1—C3—O2	5.5 (7)		

Symmetry codes: (i) −*x*+1, −*y*+1, −*z*+1.

## References

[bb1] Brandenburg, K. (2006). *DIAMOND* Crystal Impact GbR, Bonn, Germany.

[bb2] Bruker (2009). *APEX2* and *SAINT* Bruker AXS Inc., Madison, Wisconsin, USA.

[bb3] Fairuz, Z. A., Aiyub, Z., Abdullah, Z., Ng, S. W. & Tiekink, E. R. T. (2010*a*). *Acta Cryst.* E**66**, m1049–m1050. 10.1107/S1600536810030187 PMC3008138 21588477

[bb4] Fairuz, Z. A., Aiyub, Z., Abdullah, Z., Ng, S. W. & Tiekink, E. R. T. (2010*b*). *Acta Cryst.* E**66**, m1077–m1078. 10.1107/S1600536810031168 PMC3007970 21588497

[bb5] Farrugia, L. J. (1997). *J. Appl. Cryst.* **30**, 565.

[bb6] Sheldrick, G. M. (1996). *SADABS* University of Göttingen, Germany.

[bb7] Sheldrick, G. M. (2008). *Acta Cryst.* A**64**, 112–122. 10.1107/S0108767307043930 18156677

[bb8] Westrip, S. P. (2010). *J. Appl. Cryst.* **43**, 920–925.

